# The School Clinic
*Previous articles in this series have appeared in The Hospital of Oct. 14, Oct. 28. Nov. 11, Nov. 25, and Dec. 9.

**Published:** 1912-01-06

**Authors:** 


### THE SCHOOL CLINIC.*

### VI.—The Managers' Point of View.

In the majority of cases, the managers of a school approach such a question as the establishment of a local treatment centre or a school clinic with some diffidence, largely owing to the fact that the economic aspect is still so vaguely discussed.

### Managers' Powers.

Already the managers have many claims on their activities and their powers are limited. It is true they have the power to veto, but when it comes to a matter of inception, of starting such a centre the establishment of which involves a considerable initial outlay, they find that their powers are extremely limited. The blame for the non-provision of such centres must therefore not be ascribed to them, nor must it be thought that they are wanting in their sense of responsibility or unacquainted with the defects of the existing system which gives us medical inspection without adequate provision for medical treatment. Quite the contrary, in fact, is the case.

As managers they fully realise that medical supervision without the means of treating such defects as are discovered on examination and inspection is a farce. There is little merit and no sense in certifying a child to be suffering so severely from discharging ears that his inclusion in class is a source of annoyance to the other pupils and his condition a danger to himself, unless we have at the same time the power to enforce that such a child is adequately treated and made fit to resume his place in the school. At present we have no such power.

There is indeed some provision whereby the managers, if they are sufficiently interested in a case, may try to secure treatment by threatening the parents or guardians with a prosecution in court if it can be shown that the defect, untreated, is of such a nature as to damage the child permanently. Moreover we can enforce cleanliness by threatening similar prosecutions in the case of verminous children whose parents have repeatedly been warned but who have failed to cleanse themselves. But school managers are averse from taking such extreme steps, because in the majority of cases they fully realise the difficulties with which parents have to contend in order to secure medical treatment for the children.

### How Their Hands are Tied.

In the case of cleanliness these difficulties are not so great since treatment can easily be carried on at home, or, if the case is a very bad one, at the cleansing stations provided in every district. But when it comes to physical defects we recognise that there are many sides to the question, and so long as the regular school clinic, centrally situated and easily accessible, giving efficient, certain, and adequate treatment where such treatment is required, is not in existence, our hands are to a great extent tied and we can do little.

From the managerial point of view what is wanted is a succinct and reliable synopsis of the methods whereby such centres have seen secured in the districts where they exist. We want, for instance, to know what it has cost to establish a school clinic, and what are the expenses of running such an institution. At present it is understood that most school clinics are in a sense private institutions, kept up by voluntary donations. None of them, apparently, is self-supporting. The economic question, therefore, is a very important one, for no board of managers can embark on a scheme without knowing the expense.

### Independent Centres.

There can, in principle at least, be very little objection to the establishment of such independent centres of treatment in connection with a group of schools in a certain district. Their establishment at each school is another matter altogether. At present accommodation is already very cramped at most schools, and to place aside a separate room for doctor and nurse is an impossibility in the majority of cases, especially as far as the older schools are concerned. Probably the best way would be to establish the centre in a separate building in the vicinity of the school and to have a doctor and nurse in attendance at certain definite periods during the day.

### The Staff Question.

Whether the staff of the clinic is to consist of local practitioners or outside experts is a matter for the authorities to consider; the points that concern the managers are that the school clinic should be easily accessible, should give adequate treatment at the lowest possible charge, and should be efficiently supervised. The last point may be secured by the appointment of consulting experts, as advised by The Hospital, and indeed the appointment of such consultants would be welcomed by the managers as affording some means of control, for under the present system—be it said in all diffidence—the opinions of the inspectors, local doctors, and hospital experts are often so varying that the average layman is needlessly perplexed.

Treatment at such centres would be confined to simple medical defects, to cases of disease of the eye, ear, nose, and throat, and possibly to simple orthopædic cases. More serious defects could not be treated at the clinic but would have to be relegated to the hospital or the local practitioner for home treatment. A clinic on this basis, if it can be established economically, will be generally approved of, and its usefulness will soon make it welcome even to those who at the present time think it a superfluity and an unnecessary piece of extravagance.

* Previous articles in this series have appeared in The Hospital of Oct. 14, Oct. 28, Nov. 11, Nov. 25, and Dec. 9.

